# A Study Toward More Ethical Commercial Aquaculture by Leveraging Rheotaxis

**DOI:** 10.3390/ani15202961

**Published:** 2025-10-13

**Authors:** Alex Raposo, Benjamin Reading, Mike Frinsko, David L. Roberts

**Affiliations:** 1 Department of Computer Science, North Carolina State University, Raleigh, NC 27695, USA; dlrober4@ncsu.edu; 2 Department of Applied Ecology, North Carolina State University, Raleigh, NC 27695, USA; bjreadin@ncsu.edu; 3 North Carolina Cooperative Extension, North Carolina State University, Raleigh, NC 27695, USA; mofrinsk@ncsu.edu

**Keywords:** striped bass, measurement, health monitoring, computer vision, prototype design, welfare, ethics, animal–computer interaction

## Abstract

The welfare of farmed hybrid striped bass remains largely unaddressed in U.S. aquaculture, despite the species’ economic significance and the scale of production. Physical handling during grading and inspection not only causes stress and increased incidence of injury, but also results in unmarketable fish and significant financial loss for producers. To address these issues, we present a prototype system that uses directed water currents to leverage the fish’s natural rheotactic behavior, enabling directed movement between tank regions without the need for direct physical contact. Our design allows for early identification of malformed individuals, who otherwise face prolonged suffering and starvation, so they can be humanely culled. In a small pilot study, we observed that fish moved into the destination region more frequently and with less behavioral variability when exposed to a directed current, suggesting this method as a viable alternative to traditional handling. While the system requires further refinement and testing at scale, these preliminary results offer a promising step toward ethical, commercially viable, and low-stress fish sorting systems in commercial aquaculture.

## Simple Summary

Current practices in aquaculture require physical handling of fish for routine measurement and health monitoring practices. These practices are labor-intensive and risk the welfare of fish by increasing both stress and the risk of significant injury. We have developed a prototype that is part of a larger system to eliminate the need for physical handling during measurement, health monitoring, and grading of farmed fish. With this prototype, we test the viability of using innate behavior in response to directed water currents (rheotaxis) to move fish through a channel, which will serve as an observation point. In the future, data will be collected at this point to determine measures such as length, indicators of health, and the population size. Results show that rheotaxis is an effective mechanism to move striped bass through a channel and past a point of observation. Further, this result indicates potential in the area of hands-free, autonomous sorting of fish as we continue to explore more fine-grained directional control of these fish.

## 1. Introduction

### 1.1. Motivation

The state of North Carolina contributes ∼3.5 million pounds of hybrid striped bass to the 8–12 million pounds produced each year in the U.S. [1,2]. These fish play an important economic role as they are sold at market for consumption and used to stock recreational ponds [2,3]. Even with this evident economic significance, little work has been carried out to ensure the welfare of this farmed species. Most existing research focuses on minimizing stress through the use of sedatives, though these interventions carry risks of their own [4,5].

As advances in other meat production industries have led to improved welfare standards and handling practices, aquaculture is due a similar evolution. Animals used for “food, fiber, or other agricultural purposes” are excluded from the United States Animal Welfare Act (7 USC, 2131–2156), meaning that the quality of farmed animals’ welfare is governed by state laws, local laws, and producers themselves. On the federal level, there are very few regulations that enforce animal welfare standards or guidelines. Two that stand out are the Organic Food Production Act (7 USC, 6501) and the Federal Humane Slaughter Act (7 USC, 1901). The Organic Food Production Act enforces quality standards on the living conditions and feed of farmed animals, ensuring access to quality food and an environment that accommodates their natural behaviors [6]. These same affordances are not ensured for non-organic livestock at the federal level. The Federal Humane Slaughter Act, which was originally passed in 1958, outlines the conditions under which an animal can be culled [7]. Specifically, the act mandates proper, non-stress-inducing treatment of animals and that pain be prevented prior to their slaughter. The act has since been amended to provide additional protections to animals, such as the ability for inspectors to halt slaughtering if inhumane animal treatment is suspected. Although these acts alone are insufficient to ensure the welfare of farmed animals, fish are not afforded any of these protections and are excluded from all federal laws on animal welfare, including the Animal Welfare Act and the Federal Humane Slaughter Act. On the state level, there are currently no laws that protect fish welfare in the United States. While organizations lobby for regulatory changes, it is a moral imperative to find other avenues to incentivize enhanced welfare for farmed aquatic species. We hypothesize that aligning welfare enhancements with market-driven economic incentives, rather than relying on regulation, is the key. Our work aims to reduce financial loss by enabling a cost-efficient reduction in unmarketable fish.

The 2023 USDA census of aquaculture reported 3453 aquaculture farms in the US, with total sales exceeding USD 1.9 billion [8]. Among these are 968 food fish farms that amassed nearly USD 820 million in sales. Instead of proposing the impossible task of eliminating or making sweeping changes to this industry, we posit a new system that is both financially advantageous to producers and improves the welfare of farmed fish. We have identified that one major impact on farmed fish welfare is stress and the risk of injury that comes from routine aquaculture procedures [9]. Both injury and stress, typically incurred during physical handling of fish, can result in premature death.

Physical handling most frequently occurs when fish are netted to be graded, inspected, or transported from their tank. Grading, in particular, occurs at a frequency of once every 1–4 weeks, depending on the facility and growth stage of the fish [10,11]. Although precise estimates are unavailable, these processes incur substantial losses due to stress and injury. Fish subjected to regular or chronic stress also yield lower quality and unfavorably flavored market products [12]. Additionally, by facilitating an environment with sufficient access to food, the natural process of cannibalistic behaviors that would otherwise prune smaller fish is suppressed. Although this is not an immediate issue, it leads to significant size variability within a population and increased input costs as unmarketable fish remain alive and consume feed or occupy space that could otherwise be allocated to marketable fish. This variability demands grading to separate fish for sale and for size-appropriate feeding.

Common malformations in striped bass include scoliosis, pughead and accompanying jaw malformations, and swim bladder issues [13,14]. These issues, particularly cranial malformations such as pughead and jaw deformities, compromise the ability of fish to feed effectively. This is because striped bass rely on a sucking action to intake and swallow food, which cannot be achieved effectively with cranial malformations [13]. Spinal and swim bladder malformations also affect feeding by impacting swimming ability, meaning they are out-competed for access to food. Collectively, feeding difficulties can result in malformed fish being severely undersized throughout the production cycle, unmarketable, and likely suffering from starvation. This results in excess financial expenditure on unmarketable fish as they consume resources that could be better allocated. Although the degree to which malformations cause pain in fish has not yet been determined, it is clear that underconsumption or starvation due to feeding difficulties is a form of suffering that negatively impacts welfare.

To address these challenges and to improve welfare alongside improving profits for producers, we propose a system that enables fish to be moved between connected regions in a tank via a channel without physical handling. We aim to show that fine-grained control on current in enclosures is sufficiently powerful to enable contact-free sorting and grading. This advancement will allow for welfare improvements such as lower-stress and injury-free grading and for malformed individuals to be sorted out earlier in their life cycle so they may be humanely culled, using recognized humane euthanasia methods such as MS-222, or rehoused with like individuals where food competition is leveled [15]. These possibilities, based on our current system and results from our past work ([16]), will support both improved welfare outcomes for fish and financial efficiency for producers.

### 1.2. Related Work

In our preliminary work, we constructed a small-scale prototype to characterize the behavior of juvenile striped bass in response to appetitive, aversive, and combined stimuli [16]. The goal was to evaluate the capacity of different stimuli to train the fish to move from a starting region to a destination region of a tank via a submerged channel. While passing through the channel, video footage was collected for use with several computer vision techniques to determine fish lengths. During trials, the appetitive stimulus was food pellets delivered to the destination region, the aversive stimulus was a perforated wall used to slowly decrease the available space in the starting region, and the third stimulus was a combination of the appetitive and aversive stimuli simultaneously. The results of this study showed a stronger, more consistent response to the aversive stimulus; however, this method had its drawbacks. Not only did it increase the risk of injury (which did not occur during the study), but it also risked elevating stress, particularly as fish exposed to the aversive stimulus lost the ability to refuse consent. At some point, there was no longer enough space in the starting region, and the fish had to move to the destination. In effect, this approach was tantamount to manual handling. It was these limitations that motivated our current study with changes in the selected stimuli and other protocol adjustments aimed at reducing researcher influence on fish behavior and improving the quality of our results.

Previous work in the area of commercial fish transport and attracting fish is limited, emphasizing the need to explore this domain. In this work, we aim to move fish between two regions of a tank by leveraging the rheotactic response using directed water currents. Positive rheotaxis, which we simply refer to as rheotaxis, is a response to water flow of orienting and moving upstream [17]. This behavior is moderated by several biological mechanisms, including the visualization of optic flow, body motion, and vestibular cues, in addition to external factors such as current rate, water temperature, and other environmental factors. Rheotaxis plays a role in locating the source of upstream olfactory stimuli and intercepting food drift, which are integral for feeding. Most frequently, rheotaxis is used by fish to prevent downstream movement while minimizing energy expenditure [18]. The rheotactic response is amplified in warmer waters, with water temperatures up to 20 °C correlating with greater lengths and areas traveled by juvenile striped bass in a test chamber. Another study attempted to refine techniques to attract fish to a desired location using a variety of “attraction flow conditions” [19]. The study exposed fish to lateral symmetric bimodal current outflow, lateral asymmetric bimodal current outflow, and single peak current outflow. The sensing success rate, attraction success rate, reaction time, and attraction time were recorded for fish exposed to each current type. Single-peak outflow conditions were found to be most successful with an attraction rate of 82% and a sensing rate of 91%. Although both symmetric and asymmetric lateral bimodal outflow conditions resulted in sensing rates of 79% and 75%, their attraction rates were substantially lower at 39% and 32%, respectively. Although this study was conducted with Crucian carp, we used these results as a starting point for selecting flow conditions in our prototype.

Once we are able to accomplish this task of automating the movement of fish in commercial production environments, we will enable other technologies, such as computer vision, to be used for automated hands-free measurement (furthering results from our last study ([16]) by measuring in real-time), counting, abnormality detection, and sorting. Similar capabilities have been successfully implemented in studies on livestock and wild species. For example, the weights of broiler chickens have been estimated using threshold-based image segmentation, linear regression models (which achieved a relative error as low as 0.04%), and BPNN, SVR, and TF models (which were each able to explain 98% of variance in weight) [20]. Counting individuals has proven to be a challenging task in species, such as fish, that are constantly mobile. BatCount is free software that uses median-based background subtraction and a nearest-neighbor tracking algorithm to count individual bats [21]. In field studies, BatCount achieved accuracies between 50.8% and 94.8% in bat counting. Although originally designed for bats, the software is claimed to be suitable for counting other mobile species such as fish. Fish counting has been accomplished with varying degrees of success in aquaculture settings. Most recently, a 2025 study on fish counting in open environment aquaculture (different from the aquaculture tank settings our work focuses on) achieved a mean absolute error of just 1.26 using an attention-guided multi-scale feature cascade network [22].

Abnormality detection is primarily used for transmissible disease screening. One application is in poultry disease screening, which uses a combination of camera and heat sensor inputs to diagnose poultry influenza and other respiratory conditions [23]. Amidst the rise in popularity of precision livestock farming, which demonstrates mounting potential to improve animal health and welfare, as well as increase production efficiency, the demand for continued technology advancement will continue to grow [24]. Such areas of growth not only include improvements in accuracy but also expanding the realm of capabilities to include more complicated tasks, such as sorting, which have yet to be tackled. We aim to develop a system that enables such growth by leveraging the functionally relevant behavior of rheotaxis, while we stray from the nutritive reinforcement and use of aversive stimuli of our past work.

## 2. Materials and Methods

In this section, we detail the prototype and experimental designs. The prototype design detailed in this paper serves as a stepping stone towards developing a larger system to reduce financial and product loss in fish farms.

Our methods detail how we manipulate water flow to engage rheotaxis as a means to direct between locations within a tank. By successfully moving fish between two regions in response to a stimulus (water currents), we open doors for more sophisticated prototypes capable of simultaneous information retrieval and sorting. Such systems are described below in future work.

### 2.1. Prototype Construction

The primary sources of loss we seek to reduce are product loss stemming from fish stress, injury, and death from physical handling, as well as financial loss incurred from rearing malformed fish. In current aquaculture systems, fish are handled during sorting, measurement, and inspection, which occurs as often as weekly in some aquaculture facilities. Malformed fish are typically identified and sorted out via manual inspection or grading. These processes can be narrowed down into two overlapping problems: sorting and information retrieval.

Information retrieval is the process of collecting observable traits or data about individual fish. Examples include the following: size, body condition, coloration, or malformations/abnormalities. Retrieved information is often used to inform management and sorting decisions. Sorting: the process of physically separating fish into different groups based on the traits identified during information retrieval.

More specifically, fish are handled to collect some information (size, whether they appear healthy, coloring, etc.) that helps farmers decide how to sort individuals. Our long-term goal is to automate the process of identifying the desired criteria and sorting accordingly without physical handling, which we hypothesize will eliminate the aforementioned sources of loss.

**Figure 1:**
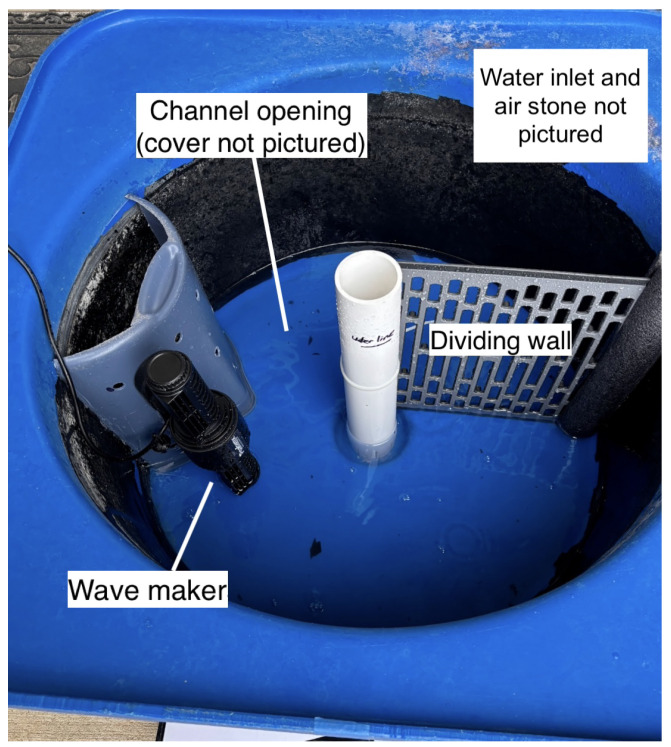
Original prototype version not used in the final trials. Contains a wide channel, 1 wave maker, and a perforated dividing wall.

As an incremental step to address the problems of sorting and information retrieval, the goal of our system is to move striped bass between two regions of a tank through a narrow passage, which we will refer to as “the channel”. The prototype shown in Figure 1 was installed in a 16-gallon cylindrical tank liner, which is placed within a larger 30-gallon tank. This tank is part of a larger system that constantly circulates filtered and temperature-controlled water. The cylindrical liner was selected because of its similarity in shape to the participants’ home tanks and dark interiors, which can have a calming effect on fish. The starting region, containing a wave maker, is separated from the destination region by a permanent dividing wall and a channel with a channel cover (the cover is not pictured in the figure). One wave maker was selected, placed near the channel entrance, and directed down the center of the starting tank region to mimic the single-peak outflow, which has been used to successfully attract fish [19]. The current rate produced was approximately 0.207 m/s.

**Figure 2:**
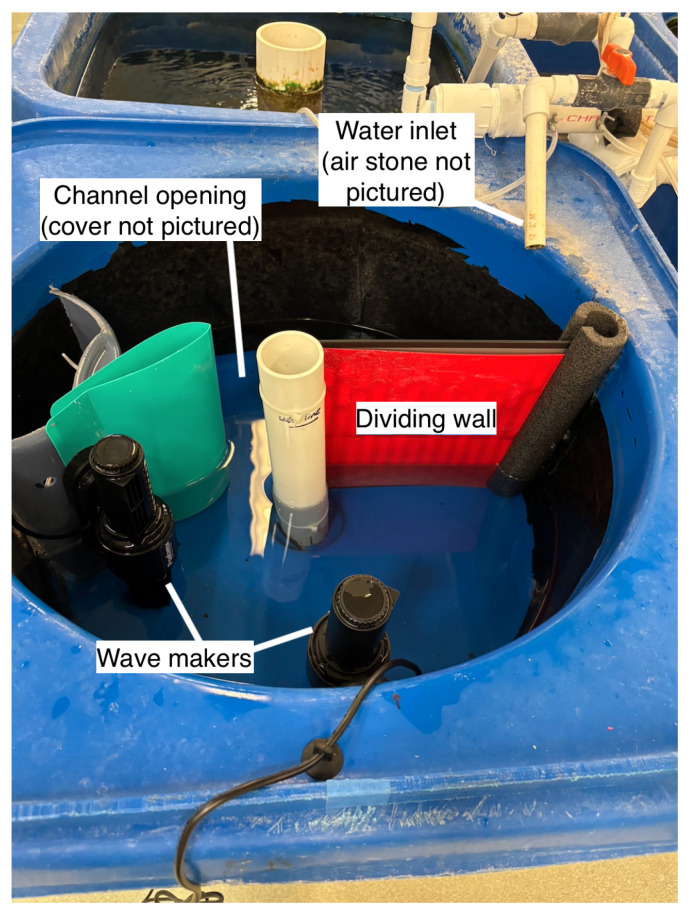
Finalized prototype used in the trials. Contains a narrow channel, 2 wave makers, and a solid dividing wall.

After running several initial experiments with a limited number of participants using the protocol described below, we made several notable adjustments to the prototype to improve performance. Instead of the desired response, participants demonstrated flow refuging behavior in the starting tank, where fish favor slower over faster current regions [17]. Additionally, when fish did exhibit the desired response of swimming into the destination region, this area was more turbulent than the low-current area in the starting region, so these fish would frequently return to the starting region and begin flow refuging behavior. To limit these types of responses, we installed an additional wave maker, placed a cover on the dividing wall, and narrowed the channel to approximate a “one-way” design. This significantly reduced the turbidity of the destination region, improved flow in the starting region, and improved the participant responses. The current rate produced was approximately 0.190 m/s. Because the approximate “one-way” design of the channel was not entirely successful in preventing participants from regressing back into the starting region, future prototypes will refine this element based on one-way fish trap entrances. Figure 2 shows the second-generation prototype used for the experimental data collection reported in this paper.

### 2.2. Interface Analysis

We will now provide a brief interface analysis using the taxonomy outlined by Freil et al. [25]. This taxonomy classifies interfaces based on the ways their intended users interact with them. An interface’s classification describes whether the interaction is trained (trained/untrained), the mode of interaction (tangible/virtual), the location of the interface device (wearable/environmental), and the direction of information exchanged during an interaction (input/output). Our system is classified as follows:

**untrained:** The interface leverages a genetically programmed behavior, rheotaxis, and does not require any learning in order for fish to respond; thus, it is untrained.
**tangible:** The interface relies on the fish’s sensory abilities to detect current flow, requiring physical interaction in order to operate; thus, it is tangible.
**environmental:** The interface exists at fixed points in the tank enclosure, and does not move to follow the fish nor is it physically coupled to their body; thus, it is environmental.
**output:** The interaction is initiated by the computing system with the purpose of conveying to the fish that it’s time to move; thus, it is an output interface.

Although we filmed interactions, the camera is not an integral part of the interface, and the visible response of the fish occurs regardless of the presence of the camera. Thus, in its current form, there is no input interface in our system. Our system was designed to adhere to this type of interaction (untrained, tangible, environmental, and output) because of the ease of use for fish participants and since limited literature suggests success in other types of interactions (such as trained ones or those relying on a wearable device).

### 2.3. Protocol

This protocol was approved by the North Carolina State University IACUC. When designing the protocol for this study, several factors were considered. Through past experience, our team has learned that the effect of a stressful event, such as an environmental change, could have lingering impacts on fish for days. These impacts include changes in appetite, activity, or even death if fish are handled improperly. To minimize these effects, we incorporated an acclimation period in our protocol. The prototype was placed in a dedicated tank in NC State University’s Grinnell Aquatic Research Facility. The tank is housed in a temperature-controlled room and belongs to a system of tanks where the salinity of the water is regularly tested, the water is constantly cycled to remove debris, and water oxygen saturation is maintained at an appropriate level using an air stone. After securing the channel cover, we filled the tank and added an airstone. After allowing the tank to sit undisturbed for 10 min, we performed a visual inspection to ensure no components were dislodged during installation. Next, we selected fish using a soft net with a preference for smaller fish due to the prototype size. Forty juvenile striped bass, spawned in the Spring of 2024 and measuring approximately 15 cm in length, were used for this experiment. Care was taken to ensure that fish had not been re-sampled. Once 5 fish were obtained, they were visually inspected before being transported in an oxygenated tank and placed in the starting region of the prototype (the region containing the wave makers). Lastly, a mesh cover was fixed over the top of the prototype as an additional precaution since jumping may occur after the initial stress of transport. This began a 4-day acclimation period. During the acclimation period, the wave makers remained powered off, we maintained a normal feeding schedule, and we visually inspected the prototype and the fish daily for signs of damage. Apart from one unexplained death in trial round 2, B, the participant fish showed no significant changes in appetite or behavior beyond day one of the acclimation period. If any fish were impacted by injury or death, they were disenrolled from the trial and treated accordingly.

After the acclimation period, we removed the channel cover and began video recording for data collect. Fish were divided into two groups: A and B.

**A**  Fish belonging to group A experienced a control period followed by an experimental period;
**B**  Fish belonging to group B experienced an experimental period followed by a control period.

The control period lasted five minutes and consisted of the wave makers being powered off (or remaining off) while fish had the freedom to move between the two tank regions. The experimental period also lasted five minutes and consisted of the wave makers in the starting region being powered on while fish had the freedom to move between the two tank regions. Throughout the experimental period, we took care to minimize the frequency with which researchers approached the prototype. This precaution was intended to reduce the impact of the researchers’ presence on the fish’s stress levels, which was a noted improvement from prior work [16]. Reversal of the order of the control and experimental periods was used to account for the potential confounding effect of novelty. This allowed us to distinguish whether the observed behavioral responses, such as the desired behavior of movement into the destination region, were driven by the introduction of the experimental stimulus (water current), rather than simply by the novelty of access to the destination region. After concluding both the control and experimental periods, the trial was complete, so we powered off the wave makers (for group A trials), performed a visual inspection of the fish, and returned the fish to their home tank.

### 2.4. Data Collection

**Figure 3:**
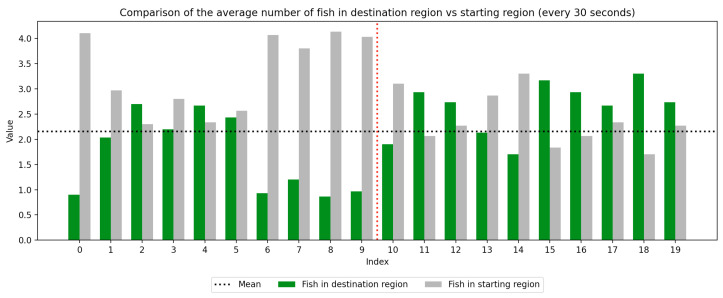
Round 1, A (Control, Experimental). Average number of fish in each prototype region every 30 s (n=5).

**Figure 4:**
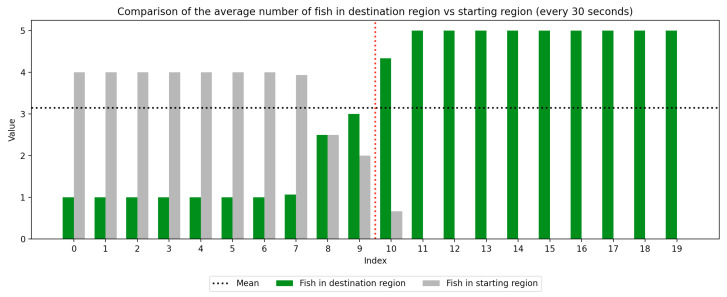
Round 2, A (Control, Experimental). Average number of fish in each prototype region every 30 s (n=5).

**Figure 5:**
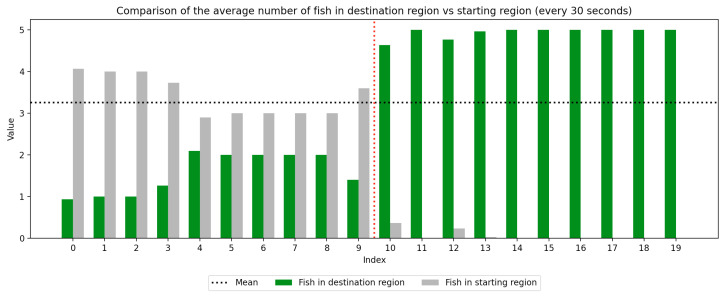
Round 3, A (Control, Experimental). Average number of fish in each prototype region every 30 s (n=5).

**Figure 6:**
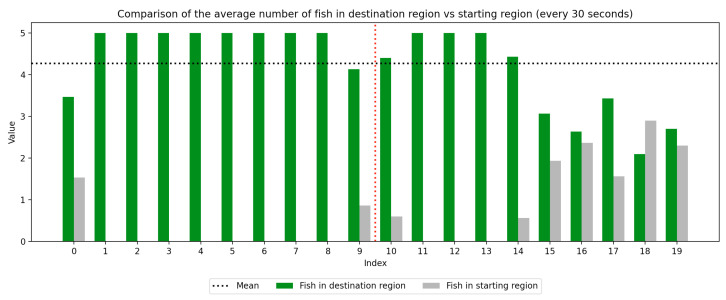
Round 1, B (Experimental, Control). Average number of fish in each prototype region every 30 s (n=5).

**Figure 7:**
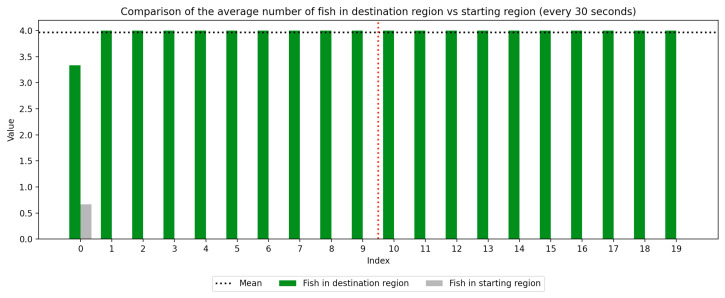
Round 2, B (Experimental, Control). Average number of fish in each prototype region every 30 s (n=4).

**Figure 8:**
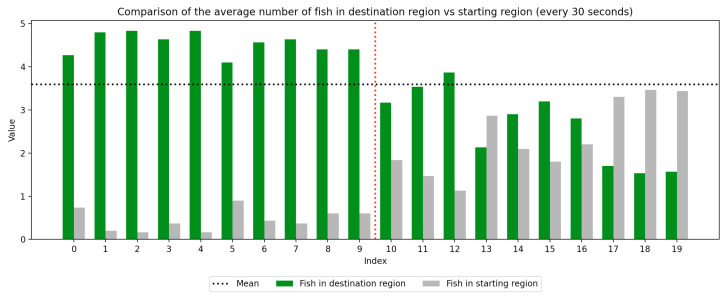
Round 3, B (Experimental, Control). Average number of fish in each prototype region every 30 s (n=5).

We recorded the experimental sessions using a GoPro Hero 12 camera mounted on a tripod above and to the side of the tank. After the sessions concluded, we transferred the video from the GoPro camera to a laptop and coded for fish location. If a fish moved between the regions at any time, this was recorded with a timestamp at the nearest second. Using this record, we were able to derive a log of the number of fish in each region at every second of the trial. We next divided the 10-min trial sessions into 30-s segments to compute the average number of fish in the regions (starting vs. destination) during each time window, which was used to visualize trends over the course of the whole trial as well as within the control and experimental periods, which are separated by a red dotted line. Results are presented in Figure 3, Figure 4, Figure 5, Figure 6, Figure 7 and Figure 8 below.

## 3. Results

Here, we present the results of experimental data collection using the second prototype, shown in Figure 2, with 40 juvenile striped bass, split into 8 trials (5 fish per trial) belonging to either group A or B. Additionally, we summarize the results of our prior work using Computer Vision to estimate fish length and weight [16].

### 3.1. Movement Analysis

During the acclimation period, many fish initially gathered near the slotted channel cover (see Section 2.1). This area allowed partial visualization of the destination region. This behavior may indicate visual investigation or exploratory desire, potentially reflecting a form of environmental curiosity or a precursor to exploratory actions. In group A trials, in which fish experienced the control period (wave makers powered off) prior to the experimental period (wave makers powered on), most individuals exhibited a recognizable “exploratory action pattern” when given access to the destination region during the control period [17]. This response can be visually characterized as the fish swimming back and forth between the two regions and swimming around the destination region. In group B trials, where the experimental period occurred prior to the control period, a similar “exploratory action pattern” was observed during the experimental period in the form of visibly heightened activity within the destination region. Exploratory actions typically diminish over time, which is consistent with our observations of both groups [17], and suggests the effectiveness of leveraging rheotaxis may increase over time. The purpose of moving fish between the two regions is not only to facilitate voluntary movement but also to position them within a camera’s field of view for information retrieval and sorting capabilities.

As we can see in these Figures, and is confirmed by the averages provided in Table 1 and Table 2, the average number of fish in the destination region was consistently higher during the experimental period than during the control period. The only exception to this finding is Round 2, B, where all participants moved into the destination region during the experiment period and remained there for the entirety of the control period. We conducted a paired sample *t*-test to compare the number of fish in the destination region across control periods to those values for experimental periods across all trials and found this difference significant (t=−38.9529, $p=4.37\times 10^{-271}$). Eight individual *t*-tests conducted on data within each trial group confirm this finding as well (p<0.05). These results indicate a successful response of exhibiting the desired behavior of moving into the destination region when exposed to the stimulus.

Next, we will analyze the variance within the control and experimental periods of both trial groups. Participants belonging to group A experienced the control period prior to the experimental period, and group B experienced the experimental period prior to the control period. Here, variance characterizes the change in the number of participants in the destination region during the time periods. By comparing the variance of the control and experimental periods, we can compare the behavior of fish during each period across study groups. As shown in Table 3 and Table 4, the average experimental variance is lower than the average control variance in both groups A and B, meaning that there was movement of the participants between the two tank regions during the experimental period than during the control period: participants tended to move to the destination region and remain there rather than move back and forth. The only round where this did not hold true was round 2, B, where all fish moved into the destination region during the experimental period and remained there for the duration of the control period. Using this observation of higher variance in the control period than the experimental period, coupled with the higher average number of fish in the destination region during the experimental period, we can conclude that fish exhibit the desired behavior of moving into the destination region and remaining there during the experimental period more than they do during the control period. These findings were consistent with the outcome of an F-test conducted to compare the variance within all control groups to that within all experimental groups, which indicates these differences are significant (F=2.2206, $p=2.22\times 10^{-16}$). Because of an unexplained death during the acclimation period of group 2, B, there were only four participants in this round while all other rounds had five participants. For this reason, the data were first normalized within each trial group before they were used for the F-test.

Taken together, these data and support analysis strongly suggest there is great potential for directed water currents to be used as an effective stimulus for moving striped bass between tank regions.

### 3.2. Automated Sizing

**Figure 9:**
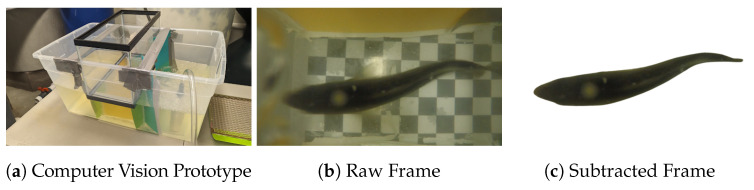
(**a**) The prototype used for collecting images for automated grading. (**b**,**c**) The raw and processed images collected using the prototype and used for estimating fish length.

In our prior work ([16]), we used the first prototype (shown in Figure 9a) to move fish between two regions of a rectangular tank via a submerged channel. This channel housed a GoPro camera that captured video footage as fish moved through the channel. From these videos, we captured still images of the fish (Figure 9b), which we used to calculate their length using computer vision. We compared two methods for this computation: a “Pixel Counting” algorithm and a neural network.

Pixel counting removes the background from images (Figure 9c), leaving only the pixels in the image that contain the fish. Using contour detection, the approach then counts the number of pixels in the longest curve on the fish’s back. We then use the number of pixels spanned by this line to estimate the length of the fish.

The neural network that we trained to predict length given images of fish consisted of two convolutional layers with 32 and 64 filters. Each layer was followed by a max pooling layer and the output was flattened. The model also contained a dense hidden layer with 64 units and the final output layer for regression. We preprocessed images using normalization and split the images into training and validation sets. The model trained over six epochs with a batch size of four, Adam for the optimizer, and mean squared error for the loss function.

To characterize the performance of these methods, and to build evidence for the potential for automated grading and sizing, we also collected the “ground truth” length measurements for a population of XX fish. Overall, we found the neural network method to be more accurate than the traditional pixel counting method; the average error for the pixel counting algorithm was 16.19%, whereas the average error for the neural network was only 5.17%. In future work, we will expand on these preliminary results to (a) reduce error and (b) incorporate disease or malformation detection.

## 4. Discussion

In Section 3, we presented data to support the feasibility of using directed current to engage positive rheotaxis behaviors to guide fish to different regions of tanks, as well as the results of a preliminary feasibility study on the use of computer vision to automatically size fish. These two sets of results are independently promising, but taken together, they form evidence to support continued research in the direction of automated sorting and grading of farmed aquatic species. Even with minimal improvement, the 5% error rate of the neural network sizing approach is plenty accurate for production, and the clear rheotactic response in the sample population indicates it is feasible to use current control to move fish to desirable areas of tanks. This capability, when combined with further development of computer vision systems, will enable touchless sorting and grading to improve welfare, reduce loss, and increase economic productivity for producers.

### 4.1. Ethics

The field of ACI has three established goals for animal welfare: improving quality of life, promoting positive effects and minimizing negative effects on animals, and facilitating inter-species or within-species communication [26,27]. Our system does not directly achieve all of these, but rather serves as a stepping stone to develop systems that make the goals of improving quality of life and promoting positive effects and minimizing negative effects on animals an achievable reality in commercial aquaculture operations. Transportation, grading, and manual handling or inspection of fish induce significant stress and pose risks of injury and death. These risks emphasize the importance of eliminating the need for physical handling to complete routine tasks in aquaculture. However, the use of the current prototype comes at the cost of confining the fish to a smaller tank than that in which they are typically housed. This design choice was made under material and practicality constraints, with the understanding that more spacious prototypes would be necessary in the future. For these reasons, our current system partially achieves the goal of improving the quality of life as a step towards eliminating the need for physical handling of fish during transportation and health monitoring activities. Full realization of this goal is possible in larger future designs. Our system meets the goal of promoting positive effects and minimizing negative effects on animals, especially those in situations where the animals have no choice in their participation. The positive effects promoted include supporting natural behaviors such as voluntary movement through rheotaxis and allowing choice and withdrawal of consent. The negative effects minimized include stress and risks of injury from physical handling and grading. Our system does not address the goal of facilitating interspecies or within-species communication, though future designs may enable a type of interspecies communication between humans and fish through early detection of conditions such as lesions and congenital malformations that may otherwise go undetected by the human eye.

### 4.2. Necessity

Given the current gaps in legal protections for fish welfare, the economic importance of the aquaculture industry, and the consequences of conventional handling practices that negatively impact both fish and farmers, the need for an ethical, non-invasive method of fish handling for information collection and sorting is urgent. As previously noted, farmed fish are excluded from all federal animal welfare laws in the U.S., including the Animal Welfare Act and the Federal Humane Slaughter Act. Without legal protection, a financially advantageous system that reduces sources of suffering and unnecessary mortality is imperative. Current common practices in aquaculture for grading, inspection, and sorting induce stress in fish and pose a significant risk for injury, leading to financial loss and compromised welfare. Furthermore, chronic stress reduces meat quality, lowering its market value and affecting consumer satisfaction. The prototypes presented in this paper leverage fish’s natural rheotactic behaviors by using directed water currents to induce voluntary movement into a specific tank region. This behaviorally driven movement allows fish to be positioned in front of a camera for information retrieval, enabling early detection of malformations and potential automated sorting without physical handling. In the absence of regulatory safeguards, the development of a technology-driven, non-invasive solution represents a crucial step toward improving both the sustainability and ethics of aquaculture operations.

In addition to the ethical benefits of a welfare-informed system for automated health monitoring and grading, such a system is also economically aligned, making it more likely to be adopted. Aside from regulatory costs, feed, fingerlings, and labor are the top 3 costs of striped bass production [28,29]. Striped bass fingerlings are juvenile fish weighing up to a few grams and are purchased from nurseries by fish farms to grow to a marketable size. As the fish grow, food and labor costs continually increase. Due to demand and market variability, once the fish have reached market size at around 1.3–1.8 kg and are ready for harvest, the high cost of rearing them can exceed their market value, preventing profit or even resulting in net loss [30]. A system that can detect and sort out unmarketable fish earlier in their life cycle would save on both labor and feed costs, though more work is needed to assess the degree to which this would financially benefit farmers.

### 4.3. Consent

This system was intentionally designed to provide a greater degree of respect for fish agency than conventional measurement and handling techniques. We aim to accommodate the fish’s abilities to give and revoke consent to participate through our design. Unlike traditional handling or sorting techniques that involve direct physical manipulation, the prototype encourages voluntary movement by leveraging rheotaxis, an innate response in many fish species to water currents. This approach allows fish to participate by engaging with their environment in a way that aligns with their natural behaviors. Additionally, this prototype avoids aversive stimuli, such as a moving wall used in our prior work [16], which could risk stress or injury. Instead, fish are given ample time to acclimate to a new environment before being exposed to a familiar stimulus. At that time, they can choose to engage, or if they choose not to engage, they can find a still area of the tank to spend time in. This design supports contingent consent, in which participants can withdraw consent at any point without consequence.

As this work was experimental, we further relied on trained aquaculture specialists to screen for signs of revoking or refusing consent. Their expertise in behavioral cues helped ensure that any signs of pain, distress, or refusal were promptly identified and respected. Any fish displaying signs of sustained distress, such as erratic swimming, suppressed appetite, or physical injuries, were removed from the prototype and returned to their home tank. By minimizing stress, enabling refusal, and incorporating animal agency, this prototype sets a new standard for ethical aquaculture technologies that prioritize consent and reduce harm.

### 4.4. Future Work

Although this study has demonstrated initial success, it is a stepping stone towards the larger-scale, more complex commercial production systems. Truly having the welfare impact we seek will require solving problems of closed-loop control and scale to illustrate how fully autonomous sizing, grading, and sorting systems can reduce product and financial loss for producers while simultaneously improving fish welfare. To further advance this welfare-conscious approach to fish handling and sorting, future efforts will focus on several key developments. One priority is designing a robust one-way channel using principles from commercial fish traps to reduce backtracking and improve directional flow. Optimizing the use of water currents using more precise analysis in response to adjustments in flow conditions is also essential. This will allow for more precise direction of fish in future iterations, particularly in setups with multiple sorting pathways. Incorporating computer vision, as discussed in Section 3.2, will enable real-time image capturing and automated analysis of traits such as size, coloration, and physical abnormalities, opening the door for a sorting system that redirects fish based on health or other market criteria. Additionally, we hope to more rigorously quantify welfare improvements by measuring indicators of acute and long-term stress for comparison to traditional methods. Scalability is another key goal, as we hope to integrate future prototypes into larger-scale aquaculture operations for testing with striped bass at a variety of developmental stages. This process, along with a more rigorous cost analysis, will enable us to better characterize the return on investment for aquaculture farmers. Expanding on this behavioral research on fish will help establish design standards for automated, ethical aquaculture production systems.

## 5. Conclusions

This study presents a promising proof-of-concept for using water currents to guide striped bass through a tank without the need for physical handling. By engaging the natural rheotactic behavior of the fish, our prototype achieves voluntary movement, reduces stress, and opens the door for low-impact sorting methods that prioritize both welfare and efficiency. The results from our initial trials suggest that this approach not only reduces behavioral variability during stimulus periods but also encourages fish to remain in the desired region longer, signaling comfort and compliance. More importantly, this system supports early detection of malformed fish, allowing for humane intervention before prolonged suffering or resource waste occurs. While limitations remain in sample size and prototype scalability, this work lays the foundation for future development of behaviorally informed, automated sorting systems that can be adapted to the realities of large-scale aquaculture. Our findings underscore the value of designing with animal agency in mind and signal a shift toward more ethical practices in fish farming, practices that support both the welfare of the animal and the long-term sustainability of the industry.

## Figures and Tables

**Table 1:** Average number of fish in the destination region during control and experimental periods for group A trials.

	Round 1, A	Round 2, A	Round 3, A	Combined
Control avg.	1.6900	1.3567	1.5797	1.5421
Exp. avg.	2.6200	4.9333	4.8678	4.1404

**Table 2:** Average number of fish in the destination region (Region B) during control and experimental periods.

	Round 1, B	Round 2, B	Round 3, B	Combined
Control avg.	3.7767	4.0000 ^1^	2.6400	3.4722
Exp. avg.	4.7600	3.9333 ^1^	4.5467	4.4133

^1^ Round 2, B was conducted with 4 participants due to an unexplained death during the acclimation period.

**Table 3:** Variance of group A trials within the study periods and overall.

	Control Variance	Experimental Variance	Overall Variance
Round 1, A	1.1644	1.1461	2.4499
Round 2, A	0.5312	0.1226	3.5299
Round 3, A	0.2989	0.3600	3.0364
Average	0.6649	0.5429	2.6454

**Table 4:** Variance of group B trials within the study periods and overall.

	Control Variance	Experimental Variance	Overall Variance
Round 1, B	0.4171	1.3045	1.1015
Round 2, B ^1^	0.2230	0	0.1124
Round 3, B	0.3691	1.3683	1.7776
Average	0.3364	0.8909	0.9972

^1^ Round 2, B was conducted with 4 participants due to an unexplained death during the acclimation period.

## Data Availability

Data from this study and several files used for analysis are publicly available on GitHub at https://github.ncsu.edu/aeraposo/striped_bass_studies.git (accessed on 1 October 2025).

## Abbreviations

The following abbreviation is used in this manuscript:

ACI	Animal–computer interaction
